# Three-dimensional (3D) culture of adult murine colon as an *in vitro* model of cryptosporidiosis: Proof of concept

**DOI:** 10.1038/s41598-017-17304-2

**Published:** 2017-12-11

**Authors:** Martha Baydoun, Sadia Benamrouz Vanneste, Colette Creusy, Karine Guyot, Nausicaa Gantois, Magali Chabe, Baptiste Delaire, Anthony Mouray, Atallah Baydoun, Gerard Forzy, Vincent Chieux, Pierre Gosset, Vincent Senez, Eric Viscogliosi, Jérôme Follet, Gabriela Certad

**Affiliations:** 1 Univ. Lille, CNRS, Inserm, CHU Lille, Institut Pasteur de Lille, U1019 – UMR 8204 – CIIL – Centre d’Infection et d’Immunité de Lille, Lille, France; 2 ISA-YNCREA Hauts-de-France, Lille, France; 3 Univ. Lille, CNRS, ISEN, UMR 8520 - IEMN, Lille, France; 4 Laboratoire Ecologie et Biodiversité, Faculté de Gestion Economie et Sciences, Institut Catholique de Lille, Lille, France; 5 Service d’Anatomie et de Cytologie Pathologiques, Groupement des Hopitaux de l’Institut Catholique de Lille (GHICL), Lille, France; 6 Faculté de Pharmacie, Univ. de Lille, Lille, France; 7 Plateforme d’Expérimentations et de Hautes Technologies Animales, Institut Pasteur de Lille, Lille, France; 8 Department of Internal Medicine, Case Western Reserve University School of Medicine, Cleveland, OH USA; 9 Department of Internal Medicine, Louis Stokes VA Medical Center, Cleveland, OH USA; 10 Department of Biomedical Engineering, Case Western Reserve University, Cleveland, OH USA; 11 Laboratoire de Biologie Médicale, Groupement des Hospitaux de l’Institut Catholique de Lille (GHICL), Lille, France; 12 Département de la Recherche Médicale, Groupement des Hopitaux de l’Institut Catholique de Lille (GHICL), Faculté de Médecine et Maïeutique, Université Catholique de Lille, Lille, France

## Abstract

*Cryptosporidium parvum* is a major cause of diarrheal illness and was recently potentially associated with digestive carcinogenesis. Despite its impact on human health, *Cryptosporidium* pathogenesis remains poorly known, mainly due to the lack of a long-term culture method for this parasite. Thus, the aim of the present study was to develop a three-dimensional (3D) culture model from adult murine colon allowing biological investigations of the host-parasite interactions in an *in vivo-*like environment and, in particular, the development of parasite-induced neoplasia. Colonic explants were cultured and preserved *ex vivo* for 35 days and co-culturing was performed with *C*. *parvum*. Strikingly, the resulting system allowed the reproduction of neoplastic lesions *in vitro* at 27 days post-infection (PI), providing new evidence of the role of the parasite in the induction of carcinogenesis. This promising model could facilitate the study of host-pathogen interactions and the investigation of the process involved in *Cryptosporidium*-induced cell transformation.

## Introduction

The *Cryptosporidium* spp. are Apicomplexan parasites of medical and veterinary importance. Currently, more than 25 *Cryptosporidium* species have been described, but the number of newly named species is increasing continuously. Among these, some are considered to be host–specific, while others exhibit a larger host range, such as *Cryptosporidium parvum* (*C*. *parvum*)^1–3^. *C*. *parvum* and *C*. *hominis* are responsible for most human cases of cryptosporidiosis, with severe diarrhea being the main clinical manifestation. Indeed, *Cryptosporidium* is considered the second cause of childhood diarrhea leading to infant mortality in Africa and Asia^4^. Unfortunately, this parasite remains one of the major causes of diarrheal disease for which no effective therapy is available^5^.

Furthermore, *C*. *parvum* has been associated with digestive carcinogenesis in different populations in humans. Epidemiological studies in Poland reported a frequency of 18% and 13% of cryptosporidiosis in patients with colorectal cancer^6^. A potential association between *Cryptosporidium* infection and bile-duct carcinoma was also suggested in children with X-linked hyper-IgM syndrome^7^. Another study reported that the risk of colon carcinoma is significantly elevated among AIDS patients presenting cryptosporidiosis. Consistent with a potential tumorigenic role of this parasite, the ability of *C*. *parvum* to induce gastrointestinal cancer has also been established in a rodent model^8–11^. *C*. *parvum* was found to induce colic adenocarcinoma in SCID mice, even with very low doses of inoculum^12^. Therefore, identifying a surrogate model for studying *C*. *parvum* pathology and induced carcinogenesis in an *in vitro* setting is extremely challenging.

While progress has been made in developing technologies for the study of *Cryptosporidium*, including the development of culture systems, animal models, and molecular genetic tools that allow the transfection of the parasite^13^, the understanding of *Cryptosporidium* pathogenesis remains problematic. Concerning efforts to develop *in vitro* models, the inability to propagate *Cryptosporidium* continuously *in vitro* has been a major obstacle to studying the cell invasion, immunological response or pathogenesis of this parasite. Most *in vitro* culture studies to date have been performed using cancer-derived epithelial cell lines and all still suffered from failure of long-term parasite spreading, low yields of oocysts and/or lack of reproducibility^14^. In particular, the most common *Cryptosporidium* culture system is based on the use of immortalized cell lines, such as HCT-8^15^. In fact, the HCT-8 cell line supports a higher rate of infection than alternative ones, without diminishing infectivity with cell age^16^. However, the two-dimensional (2D) cell culture models remain limited in the understanding of the microenvironment that contributes significantly to cell response. On the other hand, some studies have reported the development of *Cryptosporidium* in different cell-free culture systems^17^, with one major limitation in the ability to show internal ultrastructure of sexual development of the parasite. Only recently did a study confirm the gametogony and sporogony of *C*. *parvum* in cell-free cultures and describe their ultrastructure as well^18^. However, due to the absence of cells, this method is not useful for the study of the host response either to parasite infection or parasite carcinogenesis induction.

Another alternative has been the development of three-dimensional (3D) approaches. For instance, the HCT-8 organoid model has been applied in order to replicate the intestine and thus assess the human epithelial cell response to parasite infection^19^. However, this interesting model is also based on the use of transformed cells, and these cells are not able to reliably mimic the host-parasite interactions in a normal physiological way. The use of a primary culture of intestinal epithelial cells^20^ allowed *in vitro* infection by *Cryptosporidium* for up to 5 days. While this model supported *Cryptosporidium* infection better than conventional *in vitro* models, it remains limited in time.

In fact, the current challenge in systems biology is to explore the intricate dynamics that orchestrate the cellular microenvironment in which complex signaling pathways oversee the cellular phenotype in relation to tissues formation, function and pathophysiology^21^. Two-dimensional (2D) cell culture models remain limited in contributing to the understanding of the microenvironment that contributes significantly to the cell response. Therefore, it is believed that tissue culture can enable, in a more physiological manner, the response of the cell when infected. Moreover, the morphology and homeostasis of the intestinal epithelium arise from a highly regulated balance between cell proliferation, motility, differentiation and apoptosis^22^. This equilibrium is harmonized by significant crosstalk between the epithelium and adjacent cell layers^23^. Therefore, as soon as the intestinal epithelial cells are removed from the basement membrane, apoptosis is initiated within a few hours^24^. This evidence suggests that the only efficient way to study normal intestinal epithelial physiology *in vitro* may be an organ culture system, in which the epithelial cells remain in contact with the underlying mesenchyme. Consequently, the main objective of the present study was to develop a three-dimensional (3D) adult murine culture model of *Cryptosporidium* infection using colon sections from SCID mice, shown in the past to be susceptible to infection by the parasite^8,11^, and to apply this model to investigate cancer development *in vitro*. Models from primary intestinal epithelial cells or explants have been used successfully to model infection with other pathogens, such as *Candida* ^25^ or HIV^26^. In addition, while animal models remain very useful for the study of host-pathogen interactions, the advantages of finding alternative methods include the reduction in the number of animals used, the ability to obtain results quickly, decreased experimentation costs and better control of the experimental variables^27^.

## Results

### Validation of a colonic explant as a tissue culture system

#### Histological analysis

**Figure 1:**
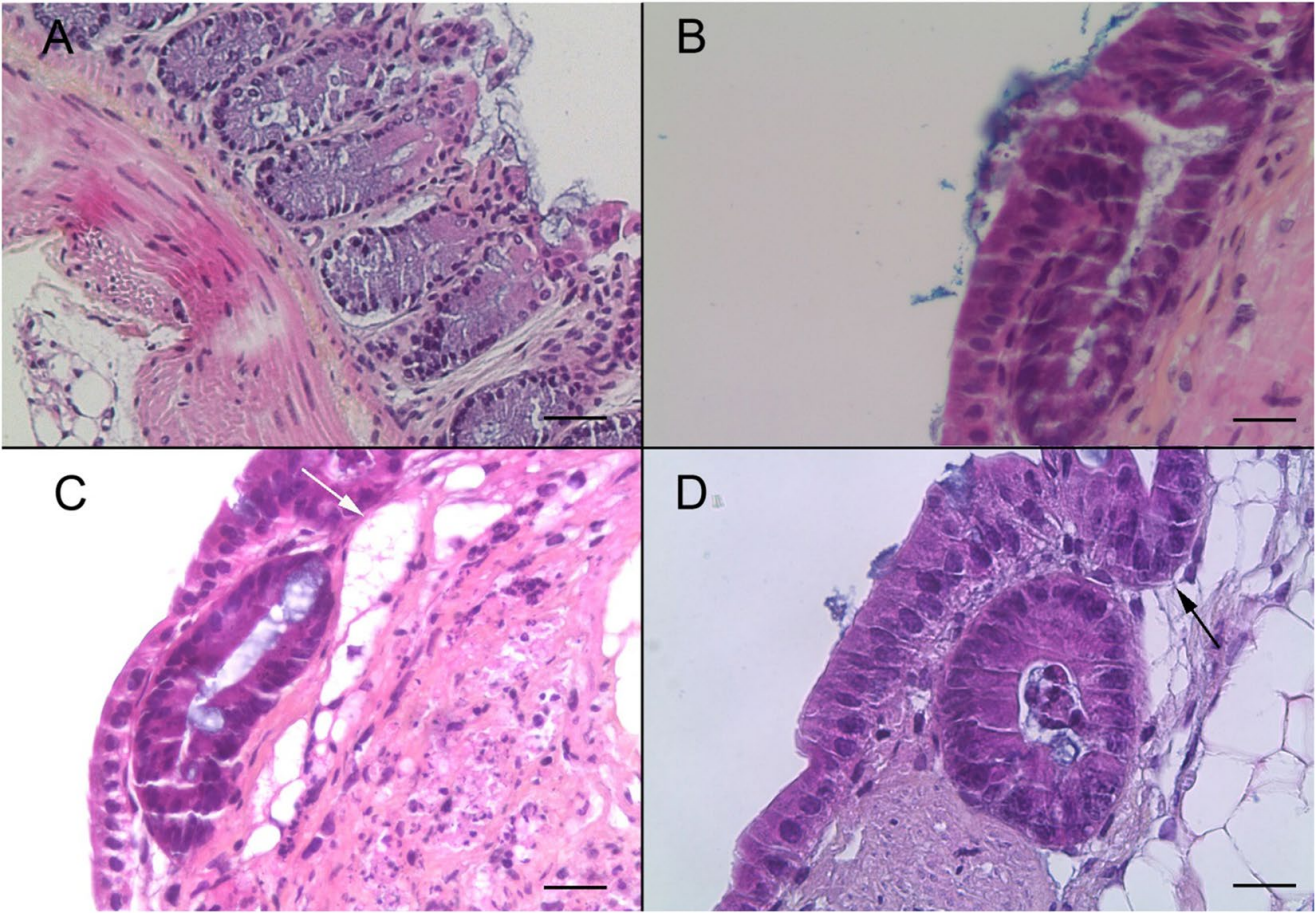
Histological validation of the murine colonic explant culture system (**A**) Hematoxylin eosin safranin (HES) staining of a normal colon section of an uninfected SCID mouse. Scale bar, 40 µm. (**B**) HES staining of a colonic explant section after 14 days of culture showing good tissue preservation with a characteristic collagen fiber and crypt-like structures. Scale bar, 20 µm. (**C**,**D**) HES staining of a colonic explant section after 21 and 30 days of culture, respectively. Tissue preservation was confirmed with a presence of high prismatic epithelium, basal position of nucleus, a well-organized basal lamina and the presence of crypt-like structures (black arrow (**D**)). Presence of large cyst-like formations (white arrow (**C**)). Scale bars, 20 µm.

In order to develop a primary intestinal model for *Cryptosporidium* and to study parasite-induced neoplasia development, firstly 18 three-dimensional tissue samples of adult SCID mouse colon were cultured on membrane inserts for up to 35 days. Mouse colonic sections obtained immediately after dissection were used as controls for immunohistochemical characterization. When the orientation of the tissue was respected and the intestinal lumen was positioned at the air-medium interface, a high prismatic epithelium covered the surface of the tissue culture and formed crypt-like structures, and the presence of microvilli was noted on the apical surface of epithelial cells. Culture explants were stopped periodically and, in 13 explants kept for 35 days, normal histological organization specific to epithelium was observed (Fig. 1A–D). This tissue preservation was characterized by nuclei located in a basal position, connective tissue showing collagen, fibroblasts, and smooth muscle cells. A well-organized basal lamina could also be detected. In addition to the crypt-like structures, large cyst-like formations could also be observed. These cysts lined by an epithelium developed inside the three-dimensional culture. No bacterial contamination was detected throughout the period of culture.

#### Cell proliferation and cytotoxicity assay

**Figure 2:**
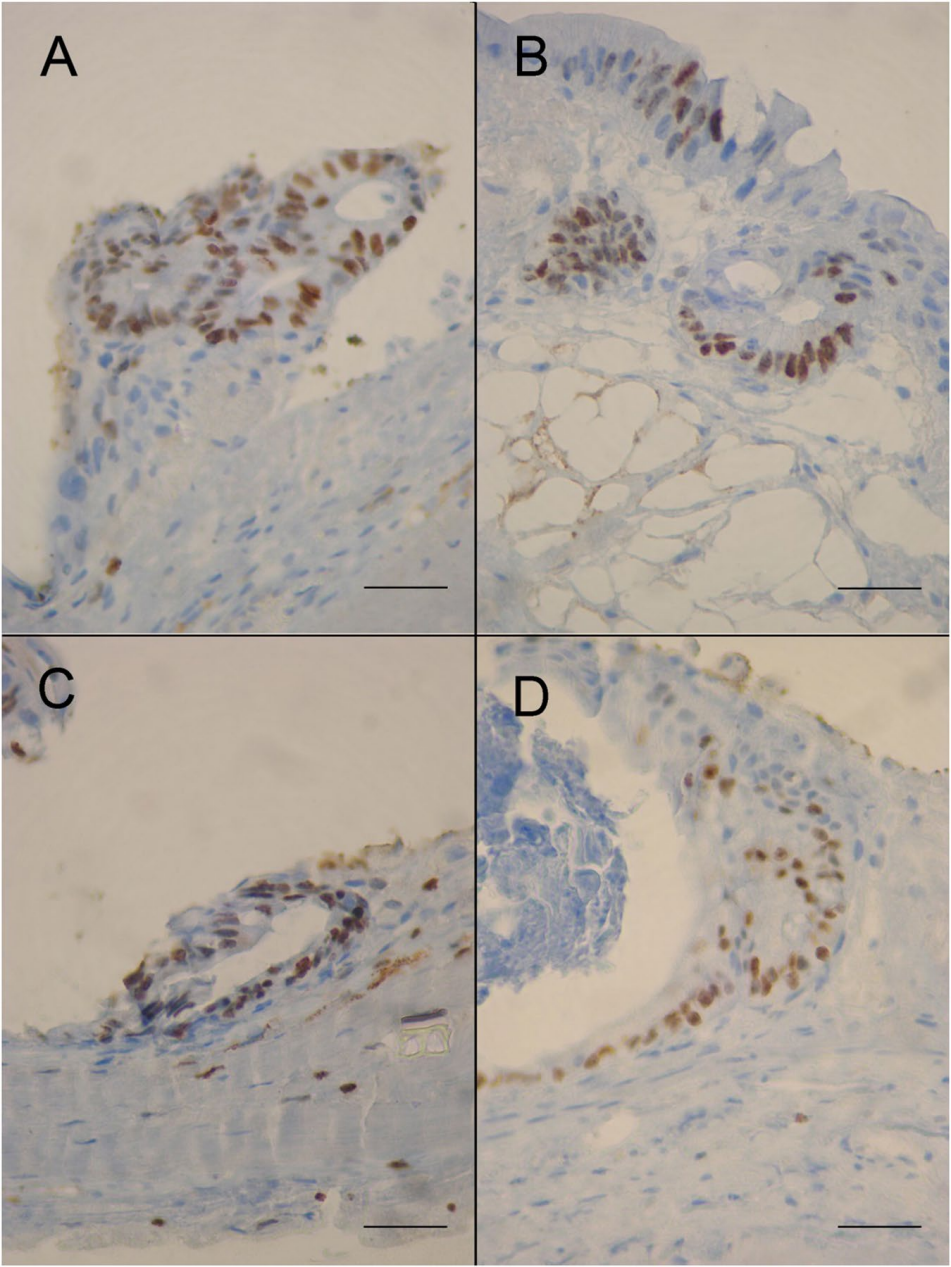
Expression of the Ki-67 marker of proliferation in the epithelium of murine colonic explants (**A–D**) Ki-67 expression is maintained within the epithelial cell layer throughout the culture period in explant sections of mouse colon after 8 (**A**), 12 (**B**), 14 (**C**) and 30 (**D**) days of culture respectively. Ki67 labeling seems to gradually diminish with increasing culture time. Scale bars, 25 µm.

Using Ki67 labeling on 5 µm thick paraffin sections, proliferating cells were identified within the epithelial cell layer (Fig. 2A–D). In parallel, cellular cytotoxicity was evaluated by measuring the rate of lactate dehydrogenase (LDH) in the culture media (Table 1). The LDH concentration was evaluated for nine samples over seven different cases (different culture periods). Of the two interactions, sample and time in culture, none was associated with a significantly low p-value. Therefore, there was no significant evidence to suggest that the LDH concentration was increased significantly. This observation led us to consider that there was no evidence of cellular cytotoxicity.

**Table 1:** Cell cytotoxicity assay throughout the period of the explant culture. (A) Mean LDH concentrations (IU/L) released into the supernatant of the explant culture until day 35 (D35). (B) Degrees of freedom, F-value and p-value of the ANOVA test.

Variable	Time in culture (days)	Number of pooled samples	Mean*	SD	Median
LDH concentration (IU/l)	12	9	53.27	35.5	45.2
	14	6	52	33.3	46.35
	26	6	57.1	32.7	42.1
	28	6	62.96	31.9	52.3
	30	3	54	38.9	39.2
	32	3	62.7	37.1	41.5
	35	3	62.2	36.9	40.5
**Source**	**Degrees of freedom**	**F-value**	**p-value**		
Subject	8	1.94	0.106		
Day	6	0.14	0.990		

*The LDH values were compared to a negative control (LDH = 50.1 IU/l) and a value measured when tissue was damaged (CN, LDH = 130.1 IU/l). Data is from minimum 3 replicates of 7 individual samples.

### Explant infection with *C*. *parvum*

#### Parasite detection in histological sections

**Figure 3:**
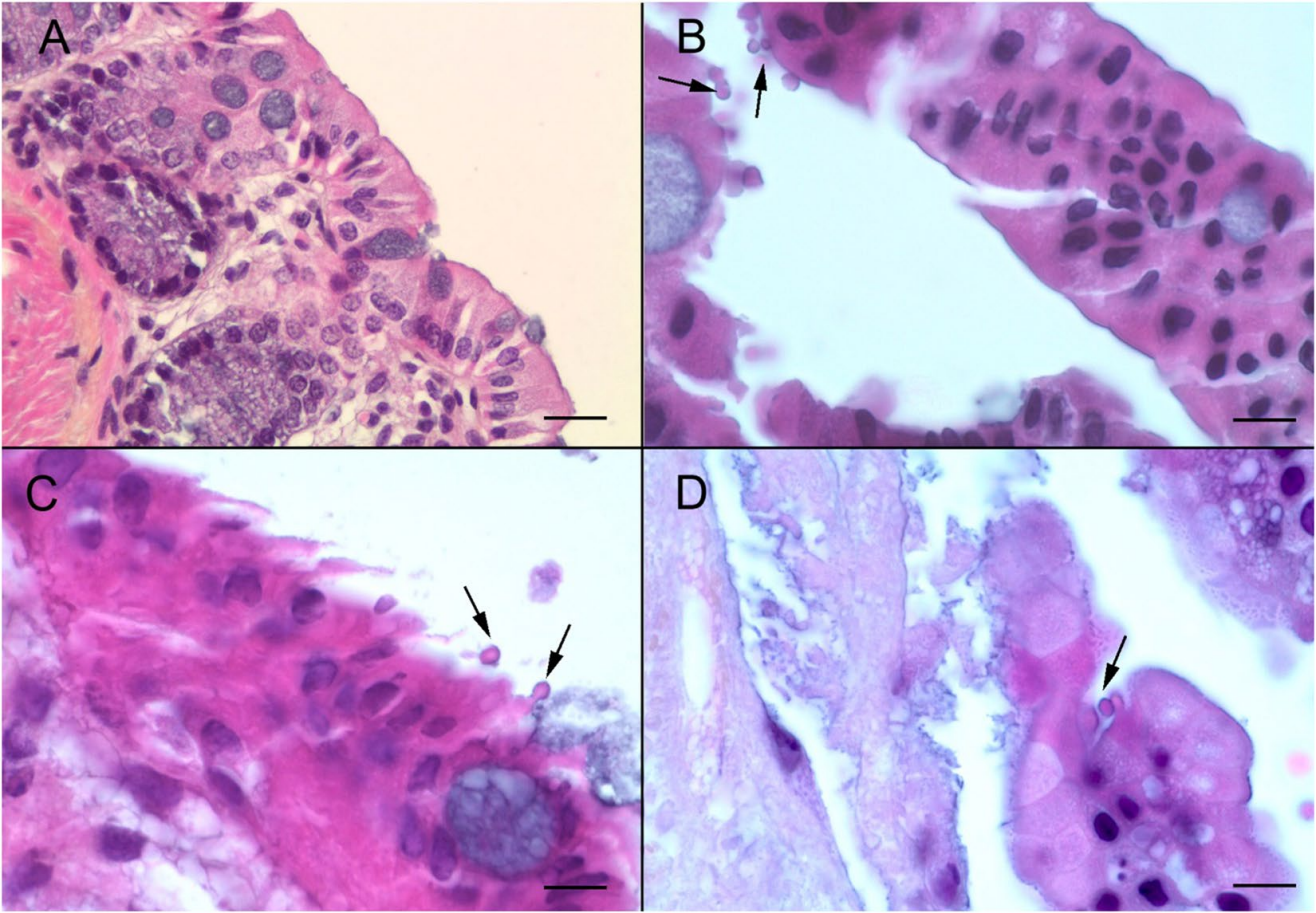
*C*. *parvum* infection of the murine colonic explant system (**A**) Hematoxylin eosin safranin (HES) staining of an uninfected SCID mouse colon section. Scale bar, 15 µm (**B**) HES staining of a colonic explant section after 12 days of culture followed of 4 days after infection with *C*. *parvum*. Developmental stages of *C*. *parvum* are observed in the apical position (arrows) within the intestinal epithelial cells. Scale bar, 10 µm (**C**) HES staining of cultured colonic explant sections after 14 days post-infection with *C*. *parvum* showing extracellular and intracellular stages of the parasite (arrows). (**D**) HES staining of cultured colonic explant sections at 22 days post-infection with *C*. *parvum* showing parasites within the intestinal epithelial cells. Scale bar, 25 µm.

After standardization of the culture system, the explant culture was prepared for the infection assay with 25 or 250 excysted *C*. *parvum* oocysts diluted in 10 µL of culture medium. It was previously reported that, after 3 days of culture, the intestinal epithelium will undergo a subsequent reorganization and will be completely renewed^28^. Based on this observation, 36 tissues were infected after 8 days in culture in order to assure complete renewal of the epithelium and tissue adaptation to the culture system. From 4 days PI and until 22 days PI, the presence of intracellular stages of *Cryptosporidium* located in an apical position was confirmed in 27 sections of infected explants stained with hematoxylin, eosin and safranin (Fig. 3). Two out of 27 showed a flattened epithelium, probably due to the explant handling. In all 27 live explants (Table 2), extracellular stages (oocysts) were also observed. No bacterial contamination was detected during the culture period.

**Table 2:** Summary of histological observations according to the initial doses of infection, the infected explant and the day of culture.

Explant identification (D = day Post-Infection)	Infective dose of *C*. *parvum*	Epithelium conservation	Presence of parasites according to microscopic observation	Presence of neoplasia
25(1)-D10	25 oocysts	Yes	No	No
25(2)-D10		Yes	No	No
25(3)-D10		No	N/A	N/A
25(1)-D12		Yes	No	No
25(2)-D12		No	N/A	N/A
25(3)-D12		Yes	No	No
25(1)-D14		Yes	No	No
25(2)-D14		No	N/A	N/A
25(3)-D14		Yes	Yes	No
25(1)-D21		No	N/A	No
25(2)-D21		Yes	Yes	No
25(3)-D21		Yes	Yes	No
25(1)-D30		Yes	Yes	No
25(2)-D30		Yes	Yes	No
25(3)-D30		No	N/A	N/A
25(1)-D32		Yes	Yes	No
25(2)-D32		Yes	Yes	No
25(3)-D32		No	N/A	N/A
25(1)-D35		No	N/A	N/A
25(2)-D35		Yes	Yes	Yes
25(3)-D35		Yes	Yes	Yes
250(1)-D10	250 oocysts	Yes	Yes	No
250(2)-D10		Yes	Yes	No
250(3)-D10		Yes	Yes	No
250(1)-D12		No	N/A	N/A
250(2)-D12		No	N/A	N/A
250(3)-D12		Yes	Yes	No
250(1)-D14		Yes	Yes	No
250(2)-D14		Yes	Yes	No
250(3)-D14		Yes	Yes	No
250(1)-D21		No	N/A	No
250(2)-D21		Yes	Yes	No
250(3)-D21		Yes	Yes	No
250(1)-D30		Yes	Yes	No
250(2)-D30		No	N/A	N/A
250(3)-D30		Yes	Yes	N/A

N/A: not applicable.

#### Parasite detection in culture medium

**Figure 4:**
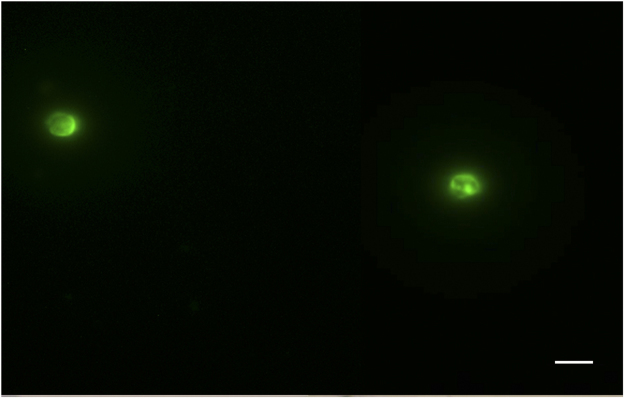
Presence of *Cryptosporidium parvum* oocysts in the medium culture pooled after 27 days PI and stained by immunofluorescence (fluorescein isothiocyanate (FITC)-conjugated anti-*Cryptosporidium*). Scale bar, 5 µm.

To identify the presence of newly formed oocysts, the culture medium collected in the first 4 days PI was discarded in order to eliminate oocysts not excysted during explant infection. Subsequently, 100 µl from each well were collected every 48 hours until 20 days PI. The collected samples were pooled together and an immunostaining was performed. Newly formed oocysts with a diameter of 5 µm were identified (Fig. 4).

#### Parasite count (or parasite number assessment or estimation) by qRT-PCR

**Figure 5:**
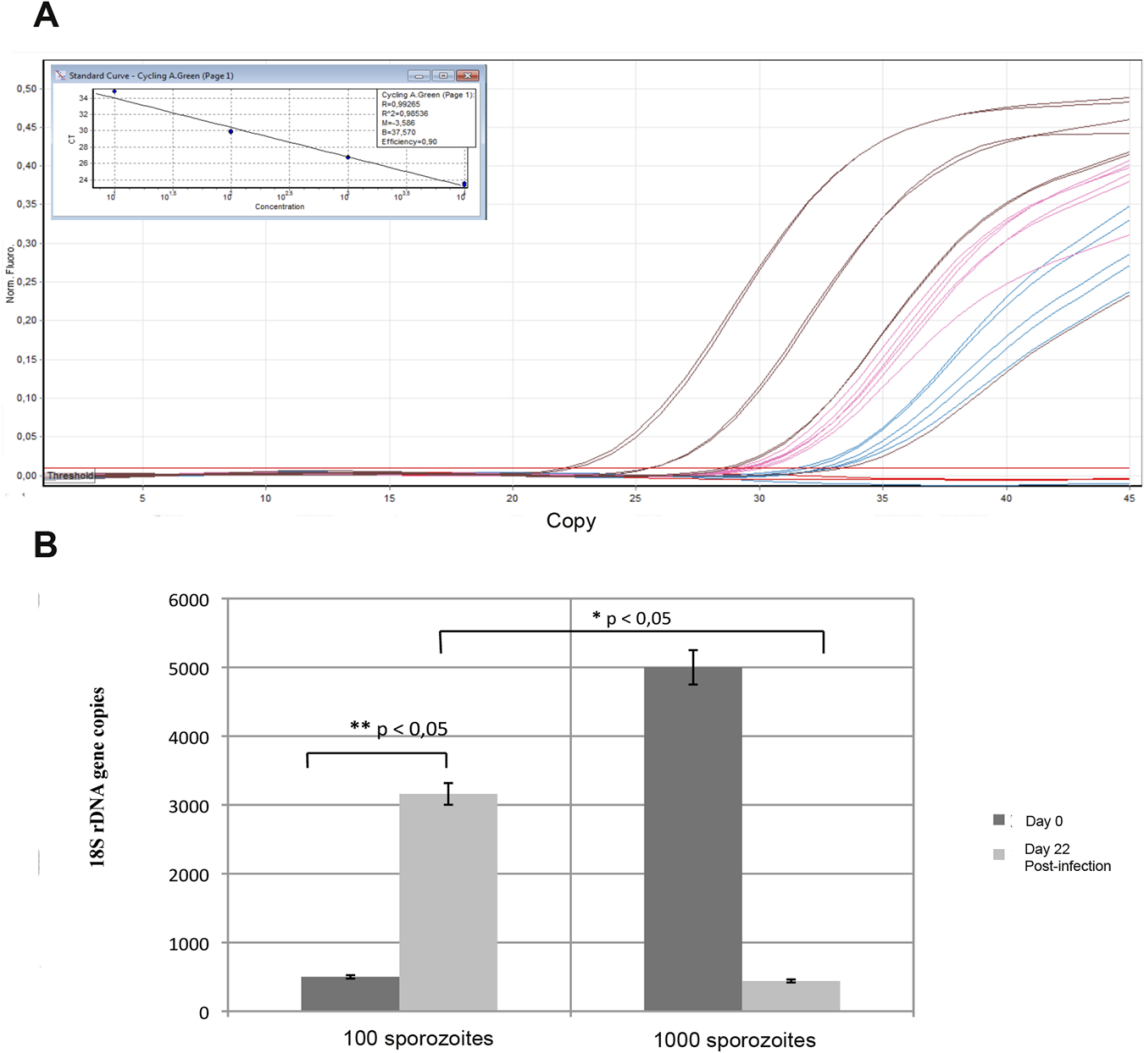
Quantification of *Cryptosporidium* 18s rDNA gene copies by qPCR in the supernatant of explants. (**A**) Standard curves corresponding to the amplification plots and (**B**) Comparison between explants infected with 25 oocysts (1) and 250 oocysts (2), respectively. Sample t-test was performed to compare values according to parasite concentrations at day 0 (D0) and 22 days PI (22D). Data is presented as mean number of copies ± SEM of three individual samples. *Indicates significant differences between the mean number of copies on the day of infection and the mean number of copies 22 days PI for tissues infected with 100 sporozoites (25 oocysts) and between explants infected with 100 sporozoites (25 oocysts) and 1000 sporozoites (250 oocysts).

Though microscopy can provide information about parasite replication, a more precise quantification of parasites was necessary to validate the model system. In order to perform quantitative analysis of *Cryptosporidium* DNA, 500 µl of the culture medium were collected every 48 hours starting at 96 h PI. All the 500 µL of renewed media from each time point were pooled. After *Cryptosporidium* DNA extraction from the pool of total collected culture volume. Final DNA was diluted in a total volume of 100 µL and 5 µL of this volume was used for qPCR. In order to evaluate the overall DNA quantity, the values obtained were then multiplied by 20 (5 µL × 20 = 100 µL). The standard curves generated showed a relationship between the Ct value and the log transformed number of copies over almost five orders of magnitude of the DNA dilution. The correlation coefficient obtained by linear regression analysis of three independent experiments was R^2^ = 0.9 (Fig. 5A). The number of *Cryptosporidium* copies was quantified by interpolation of the corresponding Ct values in the standard curves. In total, all 6 viable samples were infected by *Cryptosporidium* and a multiplication of the genomic quantity of the parasite was confirmed. The parasite load in samples infected with 25 oocysts was significantly higher when compared with samples infected with 250 oocysts (Fig. 5B).

### Study of *C*. *parvum*-induced neoplasia development

**Figure 6:**
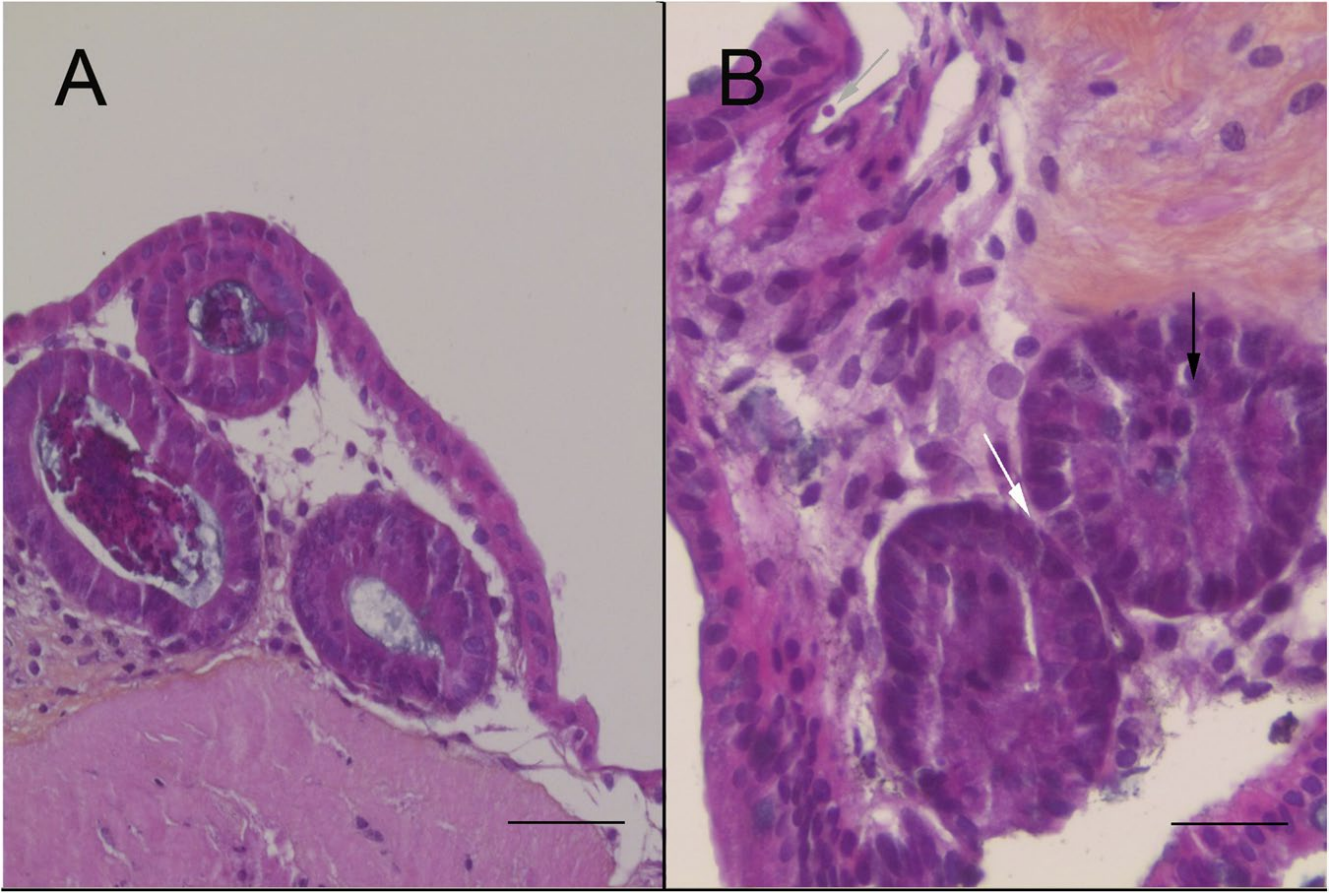
Development of neoplasia in the murine colonic explant model. (**A**) Hematoxylin eosin safranin (HES) staining of an uninfected colonic explant section showing a normal epithelial structure. Scale bar, 65 µm. (**B**) HES staining showing a low-grade intraepithelial lesion in a colonic explant after 27 days of infection with *C*. *parvum* (grey arrow) characterized by: (i) loss of cell-to-cell contact (ii) reduction of the interglandular space (white arrow) (iii) loss of nuclear polarity with slight pseudostratification (black arrow). Scale bar, 25 µm.

The morphological changes in intestinal cells induced by *C*. *parvum* were investigated by microscopic analysis of infected explants. Typical intraepithelial neoplasia of low grade characterized by loss of cell-to-cell contact was observed. The glands were slightly crowded, while having a similar size and shape and the interglandular space was reduced. Mucus droplets were also very restricted (Fig. 6B) compared to the control (Fig. 6A). The overlapping epithelium presented loss of nuclear polarity, with a slightly pseudostratification, and slightly to moderately hyperchromatic nuclei were observed. Associated with these observations, extracellular forms of *C*. *parvum* were detected (Fig. 6B).

## Discussion

In the present study, we established for the first time an explant culture model for *C*. *parvum* from adult mouse colon in an *in vivo*-like environment, and were able to maintain the infected culture for up to 35 days.

In a first step preceding infection by the parasite, a culture system maintaining the intestinal tissue from a colonic mouse on membrane inserts at the interface between air and a complex culture medium was validated. This model seems to ensure an adequate *in vitro* nutrition and oxygen supply^29^. The same conclusion was obtained in a similar experiment when pig colon was cultured at the liquid-gas-tissue interface^30^. The tissues in culture were covered by a high prismatic epithelium similar to the intestinal epithelium observed *in vivo*. After 8 days *in vitro*, the presence of microvilli was observed at the apical surface of epithelial cells. At this point, Ki-67 labeling allowed the identification of proliferating epithelial cells at the surface of the culture model that was maintained throughout the culture period. Cells proliferating along the length of the intestine throughout the culture period were closely associated with the basement membrane as the development proceeded. It has been reported that this observation could be associated with apoptosis, and it is known that this proliferating process normally plays a significant role in the mechanism by which a polarized epithelium is remodeled^31^. By using a cytotoxicity assay, we showed that no significant lysis of explant cells occurred during at least 35 days after the organotypic culture. Furthermore, all the characteristics of the epithelial barrier were preserved, such as epithelial cells, basal lamina and fibrocyte-like cells underneath the basal lamina. These observations indicated that epithelial progenitor and stem cells differentiated and that the stem cell compartment could be maintained for up to 35 days *in vitro*. After 35 days of culture, the epithelium started to flatten and fewer cells were labeled with Ki-67. Even though the fluctuation in the LDH value was not significant, we hypothesized that the tissue began to suffer after 35 days, losing the regeneration capacity and some of its characteristics such as the high prismatic organization.

Our results were compared with the data reported previously. Interestingly, similar data was obtained by Bareiss *et al*.^28^, knowing that they succeeded in preserving the living culture for only two weeks. A cultivation system similar to the one we developed here was also described by Metzger *et al*.^29^. However, these authors estimated that their system could be used for a maximum of 15 days. Moreover, even though the same scaffold membrane was used in both systems, the size of pores was slightly larger in our model. In addition, unlike these two previous systems, the culture medium was supplemented with fetal calf serum in our model, as used by Defries and Franks^32^.

We believe that the culture medium played a primary role in the preservation of the tissue and the regulation of cell proliferation, thus contributing to maintaining the epithelium for at least 35 days. Due to the fact that only 10% of FCS (fetal calf serum) was added, the medium was supplemented with other additives such as glucagon and insulin/transferrin/selenite. Additionally, KGF (keratinocyte growth factor), another additive we used, was found to enhance epithelial restitution, presumably by mediating its effects through the basolateral pole of the epithelial monolayer^33^.

Another *ex vivo* method was developed for studying mucus formation, properties, and thickness in human colonic biopsies and mouse small and large intestine explants^34^. Besides having a different medium and scaffold, two major differences were noticed between this model and the one described herein. The first was related to the tissue support, since the tissue was maintained in our study at the air-medium interface, thus preventing it from damage. In fact, only the explant with this orientation was kept alive for 35 days. The second difference was related to the removal of the longitudinal muscle layer of the intestine which was also applied by Bansal and Labruyère^35^. However, this layer was conserved in our approach to prevent additional damage to the tissue, since one of our purposes was to keep it alive for a longer period.

The results of this study highlight the important role of culture conditions, including the scaffold membrane, the position at the air-medium interface and the culture medium. Last but not least, all the previous work on colonic mice explants was done with an immunocompetent murine strain. In the present work, we used SCID mice (devoid of functional T and B cell lines) with a complementary dexamethasone treatment to complete the immune system depletion. Some previous results on cultured human fetal colon explants pointed out the involvement of the immune system in histological architecture disorganization^36^. Furthermore, results obtained by Solaymani-Mohammadi *et al*.^37^ highlighted immune-mediated alterations of the cell cytoskeleton in the intestinal epithelium.

Several other tissue culture techniques have been described, but were restricted to embryonic tissues^30,38–40^. However, the experimental manipulations of embryonic tissues for a long time are very limited, and their microenvironment remains primitive (compared to that of adult tissues). The system described herein allows easy monitoring of the experiments, respected the properties of the microenvironment and showed a high level of standardization and reproducibility.

In a second step, the feasibility of this *in vitro* system as a long-term culture model of *Cryptosporidium* was evaluated through infection experiments. Interestingly, this model was revealed to be sensitive to *C*. *parvum* infection. As expected, because of the low numbers of parasites in the initial inoculum, few extracellular stages of the parasite were observed in the early days of infection. As confirmed by microscopy, the asexual parasite forms within the cells of the digestive epithelium were abundant after 96 hours. Consistently, it has been reported that in mice infected with low inocula, the parasite excretion increased fast, reaching a mean oocyst shedding of more than 10,000 oocysts per gram of feces at 45 days PI. The few oocysts inoculated to mice probably showed a high multiplication rate in the first two weeks PI^12^. This increase in the parasite burden was also noticed in the chicken embryo tracheal infection model for *C*. *baileyi* after 72 h PI^41^. In a similar approach, human explants were used to determine gene expression in response to *Cryptosporidium* infection^42^. Ileal tissue was obtained from 3 individuals undergoing surgical procedures for unrelated noninfectious conditions. The authors were able to demonstrate that infection by *Cryptosporidium* was limited to epithelial cells and only in a minority of them. This was consistent with our HES observations showing few host cells infected with the parasite. Additionally, the supernatant obtained after 22 days PI contained newly formed oocysts positively stained with (FITC-) conjugated anti-*Cryptosporidium*. To confirm the multiplication of *Cryptosporidium* and track the infection, qPCR was performed on this sample and a high proliferation rate of the parasite was identified, particularly when the explant was infected with 25 compared to 250 oocysts. Consistently, the decrease in the mean rate of oocyst shedding after a high-challenge inoculum of parasite oocysts has been previously described in *Cryptosporidium*-infected animal models and human volunteers^11,43^. This difference in parasite multiplication could be due to competition between parasites for the limited number of intestinal cells to infect. Interestingly, the rate of multiplication of the parasite in the explant system is probably higher, considering that only the number of parasites in the culture medium was quantified here and, for logistical reasons, intracellular forms of parasites infecting the tissue were not evaluated.

In a similar approach, Castellanos Gonzalez *et al*. developed a novel method for prolonged *in vitro* cultivation of primary human intestinal epithelial cells using intact crypts^20^. The culture proliferated and remained viable for at least 60 days. However, after infection with *Cryptosporidium*, the culture was maintained only for 120 hours whereas our system culture is viable for 27 days. More recently, a 3D culture system using HCT-8 cells was adapted to the hollow fiber technology in order to provide an environment that mimics the gut by delivering nutrients and oxygen from the basal layer upward while allowing separate redox and nutrient control of the lumen for parasite development^5^. Using this technique, a high parasite multiplication was maintained for more than 6 months, producing approximately 1.10^8^ oocysts/ml on day 1, compared with a production of 1.10^6^ – oocysts/ml after 48 h in two-dimensional cultures. This system provides a unique method for high parasite production and continuous propagation, but it requires specialized equipment and is not easily scaled to be used as an experimental system for drug screening. Another limitation is that this culture process takes place in the “black box” structure that makes it difficult to observe the host-parasite interaction.

Because of the specific physiology and more complex cell organization of primary tissues, explant cultures may have a closer resemblance to the tissues observed *in vivo* ^44^. Recently, a 3D human intestinal model system was able to support *C*. *parvum* infections^45^. The 3D intestinal model system could be stably maintained for at least eight weeks in culture^46^. Even though this model has specific features usually absent in 2D models, it remained dependent on transformed cells such as Caco-2 cells. The system supported the infection for at least 15 days, whereas the system described herein supported the infection for at least 27 days.

On the other hand, the ability of *C*. *parvum* to induce gastrointestinal neoplasia has been established in a rodent model^8^. Additionally, it was found for the first time that the alteration of signaling pathways, such as the Wnt signaling pathway, and host cell cytoskeleton network, seem to be major events during the development of *C*. *parvum*-induced neoplastic processes^47,48^. Then, one of the goals of this study was the identification of a model for studying *C*. *parvum*-induced carcinogenesis. By infecting explants with the parasite, lesions corresponding to intraepithelial neoplasia were observed *in vitro* at 27 days PI, providing new evidence of the role of the parasite in the induction of carcinogenesis at least in experimental models. To our knowledge, this is the first description of a low-grade neoplasia induced after parasite infection in a 3D culture method. The aim of future studies will be to explore mechanistic insights of the *C*. *parvum*-induced neoplasia using this system that permits real-time monitoring of host-parasite interactions.

In summary, we described for the first time the development and characterization of a specialized 3D culture system for *C*. *parvum* from adult SCID mouse colon. In this system, the parasite completed its life cycle and produced newly formed oocysts. Compared to traditional 2D cultures, this system could be useful to investigate the time-dependent cellular mechanisms and factors involved in the regeneration and degeneration of host cells and a new and effective *in vitro* culture model for *C*. *parvum*, and perhaps for other intestinal pathogens. To our knowledge, this is the first 3D *C*. *parvum* continuous culture system modeling the physiopathological conditions *in vivo* thanks to the incorporation of the structural and mechanical properties that define the *in vivo* microenvironment^49^. Furthermore, the development of colon neoplasia was reproduced in the present study by co-culturing the explants with the parasite. Since the explant retained intestinal characteristics, the method described herein should provide an improved tool for studying host-parasite interactions in a microenvironment highly similar to *in vivo* conditions. In conclusion, this system could also be helpful in reducing the number of animals used in experimentation models. Finally, since one of the reasons for the lack of treatment for *Cryptosporidium* is the absence of a suitable culture system, our model could provide a new alternative to drug screening. In addition, this methodology could have wide applicability to the investigation of infectious inflammatory and neoplastic diseases.

## Methods

### Animals

The animals were kept in aseptic conditions in an isolator and were regularly inspected to assess microbial and parasitological infections (including *Helicobacter* spp.). 9 SCID mice were administered 4 mg/L of dexamethasone sodium phosphate (Dex) (Merck, Lyon, France) via drinking water as previously described^8^ two weeks before euthanasia by carbon dioxide inhalation for tissue culture experiments. Experiments were conducted in the animal facility (PLETHA Pasteur) at the Institut Pasteur de Lille (research accreditation number A59107). The animal protocols were approved by the French regional ethics committee (approval number CEEA 112011). All methods were performed in accordance with the relevant guidelines and regulations.

### Colon explant preparation

The tissue was prepared as previously described by Bareiss *et al*.^28^ with some modifications. Before culture, the colon recovered from the animals was dissected and cleaned of fecal contents with cold Hank’s Balanced Salt Solution supplemented with penicillin (100 U/ml), streptomycin (100 µg/ml) and metronidazole (50 µg/ml). After this cleaning process, the tissue was opened along its length and cut into 12 mm^2^ pieces. The explants were then transferred to uncoated membrane inserts (Millipore CM, 0.45 µm pore size) and positioned in the respective well of a six-well plate. Each well was loaded with 1 ml medium consisting of HEPES buffered DMEM/F12, 10% fetal bovine serum (FBS), penicillin (100 U/ml), streptomycin (100 µg/ml), L-glutamine (2 mM), insulin/transferrin/selenite mix (1:100), Albumax (1 mg/ml), hydrocortisone (1 µm), glucagon (14.3 nM), 3,3′,5′-triiodo-L-thyronine (1 nM), ascorbate-2-phosphate (200 µM), linoleic acid (20 µM), estradiol (10 nM) and keratinocyte growth factor (50 ng/ml). The explants were cultured in a humidified incubator at 37 °C and 5% CO_2_ for up to 4 weeks. The culture medium was renewed every 48 hours. For each culture condition and time point stop, the experiment was performed three times. In order to prevent bacterial contamination, medium culture was filtered beforehand through 0.22 µm. The absence of bacterial contamination was screened by testing the medium culture renewed every 48 hours by plating onto culture media (Trypticase soy), at least 72 hours at 37 °C. This process was performed throughout the culture period for every explant.

### *C*. *parvum* oocyst counting and concentration determination

*C*. *parvum* oocysts Iowa (purchased from Waterborne™, New Orleans, LA) were stored in phosphate-buffered saline (PBS) with penicillin, streptomycin, gentamycin, amphotericin B and 0.001% Tween 20 at 4 °C until use. Oocyst viability and the estimation of the excitation rate were assessed by a protocol as previously described^50^. PBS complemented with 0.25% (wt/vol) trypsin and 0.75% (wt/vol) taurocholic acid were used to incubate the oocysts for 1 h at 37 °C. Subsequently, 500 events corresponding to intact or empty oocysts were counted under DIC optics (Nomarski) with a Nikon 80i microscope (Nikon, Tokyo, Japan) at 630 × magnification. The excystation rate was calculated using the equation: (number of empty oocysts/total intact and empty oocysts) × 100.

The infectivity assay used, was previously developed by Keegan *et al*.^51^. Briefly, after a washing step by centrifugation at 1800 g for 20 min at room temperature, the oocysts were incubated in acidified water (pH 2.4) containing 0.025% (wt/vol) trypsin at 37 °C for 20 min to trigger excystation. After a second centrifugation step at 1800 g for 10 min, the oocysts were suspended in a maintenance medium. The maintenance medium consisted of RPMI 1640 medium with 2 mM l-glutamine, 15 mM HEPES buffer, 23 mM sodium bicarbonate, 5 mM glucose, 0.5 µM folic acid, 7 µM 4-aminobenzoic acid, 0.1 µM calcium pantothenate, 50 nM ascorbic acid, 1% (vol/vol) heat-inactivated fetal calf serum, 210 µM gentamycin, 170 µM streptomycin and penicillin (105 U/L). During the entire process, all solutions were filtered at 0.22 µm.

### *C*. *parvum* explant infection

Based on the excystation rate, the volume needed to inoculate the explant was calculated. After 8 days of culture, new explants were infected with either 100 or 1,000 sporozoites (corresponding to 25 or 250 oocycts, respectively) of *C*. *parvum* contained in 10 µl of the maintenance culture medium that was pipetted onto each well. For the negative control, 10 µl of this medium were added to the tissue culture. The culture medium was renewed every 48 hours and bacteriological controls were also performed as explained above (culture preparation section).

### Histological analysis and immunohistochemistry

The cultured explants were stopped periodically after 8, 10, 12, 14, 16, 21, 30 and 35 days after culture and then fixed in 10% formalin, and embedded in paraffin. Sections of 5 µm thickness were stained with hematoxylin and eosin (Leica Autostainer-XL, Rueil-Malmaison, France) or deparaffinized, rehydrated through serial dilution of alcohol and washed in PBS (pH 7.2) for immunohistochemical analysis. Lesions at different sites were scored as previously^12^. Sections were examined using a Leica DMRB microscope equipped with a Leica digital camera connected to an Imaging Research MCID analysis system (MCID software, Cambridge, United Kingdom).

### Cell proliferation assay (Ki-67 labeling)

The expression of Ki-67 in the intestinal epithelium was assessed in 5 μm-thick sections using a monoclonal rat anti-mouse Ki-67 antibody (dilution 1:25) (M7249, Dako, Denmark), as previously described^11^ and following the procedure recommended by the supplier.

### Cell cytotoxicity assay

The LDH was used as a marker of tissue breakdown and/or cell viability. Then 250 µL of the culture medium was centrifuged at 10,000 g for 5 min. The supernatant was recovered and tested for the quantitative determination of LDH activity using the UniCel® DxC 600/800 system. Briefly, this system was based on an enzymatic method that measures LDH activity using the LD-P (lactate dehydrogenase reagent, pyruvate → lactate) reagent. In the reaction, LD-P catalyzes the reversible reduction of pyruvate to L-lactate with the concurrent oxidation of reduced β-nicotinamide adenine dinucleotide (NADH) to β-nicotinamide adenine dinucleotide (NAD). The system monitors the change in absorbance at 340 nm. This change in absorbance is directly proportional to the activity of LD-P in the sample and is used to calculate and express the LD-P activity.

### Real-time quantitative PCR (qPCR) assay

500 µl of the medium collected every 48 h was used for qPCR starting at 4 days PI. For each well, the culture medium recovered throughout the culture was pooled. The total volume was centrifuged and a DNA extraction was performed after a step of proteinase K digestion overnight, using the NucleoSpin tissue kit (Machery Nagel, Duren, Germany) following the manufacturer’s instructions. Real-time quantitative PCR (qPCR) assays were performed using a *Cryptosporidium* TaqMan assay^52^. The qPCR was designed to detect the presence of *Cryptosporidium* DNA and amplified a DNA fragment located in the 18S rDNA gene (GenBank accession no. EU675853.1, positions 33 to 211). The forward (5′CATGGATAACCGTGGTAAT3′) and reverse (5′TACCCTACCGTCTAAACTG3′) primers were designed to amplify a 178 bp fragment. A TaqMan probe homologous to a conserved region of the sequence (Pan-crypto, FAM-CTAGAGCTAATACATGCGAAAAAA-MGB-BHQ [FAM, 6-carboxyfluorescein; MGB, minor-groove-binding ligand; BHQ, black hole quencher]) was designed to detect *Cryptosporidium*. Each qPCR was performed in a 25 µl reaction mixture containing 1X of LightCycler 480 Probes Master 2X, 200 nM of each *Cryptosporidium* primer, 10 µM of the *Cryptosporidium* probe and 5 µl of the DNA sample. The qPCR reactions were performed on a Rotor-Gene 6000 instrument (Corbett Research, Qiagen, France) and included an initial denaturation at 95 °C for 15 min followed by 49 cycles of denaturation at 95 °C for 15 s and annealing/extension at 60 °C for 1 min. Fluorescence acquisition was performed immediately following each annealing/extension step. All samples were measured in triplicate in each assay and negative controls without template were included in each qPCR run. Amplification data was obtained from the Rotor-Gene 6000 software.

### Immunomagnetic separation (IMS) and fluorescence immunoassay (FI)

The detection and quantification of the oocyst shedding from a pool of culture medium obtained between 48 h PI and until the end of the culture was performed by IMS using Dyna-beads as previously described^11^. Then, 10 µl of oocyst suspension was placed on immunofluorescence slides and labeled with a fluorescein isothiocyanate (FITC)-conjugated anti-*Cryptosporidium* spp. monoclonal antibody (Cellabs, Brookvale, New South Wales, Australia).

### Statistical analysis

To estimate whether the LDH concentration changes between measures performed at different time intervals, the repeated-measures ANOVA test was used which is defined as the measurement of the same characteristic on each case or subject at several different times or under several conditions^53^. In this study, the null hypothesis (H_0_) was that the LDH concentration means were equal for all explant samples and across the experimentation time. The alternative hypothesis (H_A_) was that at least one LDH concentration mean would be different. The LDH concentration was considered the response. The day and the subject were considered the fixed and random factors respectively. If the p-value > 0.05, H_0_ was accepted and H_A_ rejected. For each test, we planned to report the degrees of freedom (DF), the F and p values. To compare the mean LDH concentration of each subject to the reference concentration of 50.1 (IU/L), a two-tailed one-sample T-test was performed. To account for multiple comparisons, the Bonferroni correction for the five comparisons, which renders a p-value threshold of 0.01 was used. To compare the mean genomic quantity of the 18s rDNA after 22 days of infection and according to the infective doses, a two-tailed one-sample T-test was exectued. All statistical analyses were performed using Minitab® version 17.3.1 (Minitab Inc., State College, Pennsylvania).
